# Genome-Wide Association and Genomic Prediction for Stripe Rust Resistance in Synthetic-Derived Wheats

**DOI:** 10.3389/fpls.2022.788593

**Published:** 2022-02-24

**Authors:** Zahid Mahmood, Mohsin Ali, Javed Iqbal Mirza, Muhammad Fayyaz, Khawar Majeed, Muhammad Kashif Naeem, Abdul Aziz, Richard Trethowan, Francis Chuks Ogbonnaya, Jesse Poland, Umar Masood Quraishi, Lee Thomas Hickey, Awais Rasheed, Zhonghu He

**Affiliations:** ^1^Department of Plant Sciences, Quaid-i-Azam University, Islamabad, Pakistan; ^2^Crop Sciences Institute, National Agricultural Research Centre (NARC), Islamabad, Pakistan; ^3^Institute of Crop Sciences, CIMMYT-China office, Chinese Academy of Agricultural Sciences (CAAS), Beijing, China; ^4^Crop Disease Research Institute, NARC, Islamabad, Pakistan; ^5^National Institute for Genomics and Advanced Biotechnology (NIGAB), National Agriculture Research Center (NARC), Islamabad, Pakistan; ^6^Plant Breeding Institute, School of Life and Environmental Sciences, The University of Sydney, Sydney, NSW, Australia; ^7^Grains Research and Development Corporation, Kingston, ACT, Australia; ^8^Department of Plant Pathology, Kansas State University, Manhattan, KS, United States; ^9^Queensland Alliance for Agriculture and Food Innovation, The University of Queensland, Saint Lucia, QLD, Australia

**Keywords:** GWAS, GBS, stripe rust (*Puccinia striiformis* Westend), synthetic hexaploid derived wheat, haplotype GWAS

## Abstract

Stripe rust caused by *Puccnina striiformis* (*Pst*) is an economically important disease attacking wheat all over the world. Identifying and deploying new genes for *Pst* resistance is an economical and long-term strategy for controlling *Pst*. A genome-wide association study (GWAS) using single nucleotide polymorphisms (SNPs) and functional haplotypes were used to identify loci associated with stripe rust resistance in synthetic-derived (SYN-DER) wheats in four environments. In total, 92 quantitative trait nucleotides (QTNs) distributed over 65 different loci were associated with resistance to *Pst* at seedling and adult plant stages. Nine additional loci were discovered by the linkage disequilibrium-based haplotype-GWAS approach. The durable rust-resistant gene *Lr34/Yr18* provided resistance in all four environments, and against all the five *Pst* races used in this study. The analysis identified several SYN-DER accessions that carried major genes: either *Yr24/Yr26* or *Yr32*. New loci were also identified on chr2B, chr5B, and chr7D, and 14 QTNs and three haplotypes identified on the D-genome possibly carry new alleles of the known genes contributed by the *Ae. tauschii* founders. We also evaluated eleven different models for genomic prediction of *Pst* resistance, and a prediction accuracy up to 0.85 was achieved for an adult plant resistance, however, genomic prediction for seedling resistance remained very low. A meta-analysis based on a large number of existing GWAS would enhance the identification of new genes and loci for stripe rust resistance in wheat. The genetic framework elucidated here for stripe rust resistance in SYN-DER identified the novel loci for resistance to *Pst* assembled in adapted genetic backgrounds.

## Introduction

Stripe or yellow rust caused by an obligate pathogen *Puccinia striiformis tritici* (*Pst*) is a major threat to wheat production and grain quality. Wheat yield losses in different regions of the world up to 25% have been reported and this can climb to 80% when infections occur early in the crop season (Solh et al., 2012). Recently, stripe rust epidemics have damaged wheat production in many wheat growing countries and regions including Australia, Ethiopia, China, United States, Europe, South Africa, and South Asia (Milus et al., 2006; Chen, 2007; Wellings, 2011). Since Airborne *Pst* urediniospores can migrate to other regions of the world using the climatic system termed the “Western Disturbance,” thus, spreading new races. The Western Disturbance caused the spread of the (*Pst) Yr9* virulent race in the Indian Subcontinent and Nepal from the East African highlands between 1985 and 1997. In the past decade, virulence for *Yr*27 caused epidemics in Pakistan and India on the commonly growing mega cultivars, Inqlab-91, and PBW-343, respectively (Duveiller et al., 2007).

Rust resistance, like other fungal diseases, can be controlled by fungicide and resistant cultivars. However, the use of a fungicide is associated with a high cost and is hazardous to the environment. Therefore, deploying resistant cultivars is environmentally friendly and particularly inexpensive for wheat growers. To date, more than 83 *Pst* resistance genes (*Yr1–Yr83*) have been catalogued in wheat and its wild relatives (Maccaferri et al., 2015; McIntosh et al., 2016). These are predominantly race-specific major genes, which interact with the pathogen according to the gene-for-gene model and produce hypersensitive reactions. This type of resistance is usually short lived when deployed in large areas; the evolution of new pathotypes of the pathogen population leads to a resistance breakdown. Virulence on *Yr2, Yr6, Yr7, Yr8, Yr9, Yr17*, and *Yr27* are examples of major gene resistance breakdown. It is essential that new sources of resistance are found and deployed to keep ahead of pathogen changes. However, minor genes or adult plant resistance (APR) genes are an alternative for major genes and provide a quantitative resistance that is often race non-specific and durable against various pathotypes.

Wheat breeders often rely on current or old varieties as a source of resistance, however, wheat wild relatives can also provide a useful source by direct recombination, bridge crosses, or including the development of synthetic wheats (Ogbonnaya et al., 2013). Within the wheat primary gene pool, considerable genetic variation exists in *Aegilops tauschii* and *T. turgidum* for resistance to both biotic and abiotic stresses (Halloran et al., 2008). The introgression of this genetic diversity through the development of synthetic hexaploid wheat (SHW) that can be directly crossed to adapted hexaploid wheat is one such strategy. Hexaploid wheat (SHWs) are known as primary synthetics and are generally obtained by artificially crossing of durum wheat (*T. turgidum*) and *Ae. tauschii.* These SHWs have been shown to carry genetic variation for resistance to numerous biotic and abiotic stresses (Mujeeb-Kazi et al., 1996; Ogbonnaya et al., 2013). The yellow, leaf, and stem rust resistance genes *Yr28, Lr21, Lr22, Lr32, Lr39, Lr41, Sr33, Sr45*, and *Sr46* were derived from *Ae. tauschii*, and the *Sr* genes were subsequently shown to be resistant to the highly virulent *Ug99* race (Cox et al., 1995; Zegeye et al., 2014; McIntosh et al., 2016).

Genome-wide association studies (GWAS) are used to associate the genetic loci with phenotypic diversity (Huang and Han, 2014). This method combines a comparatively large portion of natural diversity in a species and localizes marker-trait associations to much shorter genomic regions because these diversity panels incorporate many more historical recombination events than classical recombinant inbred lines and doubled haploid populations (Nordborg and Weigel, 2008). The GWAS has proven to be a powerful tool for genetic analysis in wheat. It has been successful in identifying the genomic regions and markers for resistance to stripe rust in synthetic hexaploid wheat (Zegeye et al., 2014; Bhatta et al., 2019), global landraces collections (Jordan et al., 2015), Ethiopian durum wheats (Liu et al., 2017c), advanced lines derived from exotic crosses (Ledesma-Ramírez et al., 2019), Chinese wheat landraces (Long et al., 2019), global spring wheat collection (Maccaferri et al., 2015), global winter wheat collection (Bulli et al., 2016), US Pacific Northwest winter wheat (Naruoka et al., 2015; Liu et al., 2018), spring wheat (Muleta et al., 2017a), CIMMYT nurseries (Juliana et al., 2017), Afghan wheat landraces (Manickavelu et al., 2016), Ethiopian bead wheat (Muleta et al., 2017b), emmer wheat (Liu et al., 2017b), North American elite spring wheat (Godoy et al., 2017), elite ICARDA wheats (Jighly et al., 2015), diverse spring wheat (Kankwatsa et al., 2017), global landraces collection (Pasam et al., 2017), and elite durum wheat (Liu et al., 2017a).

Genome-wide prediction also referred to as genomic selection or genomic prediction is a technique to improve the selection accuracy and has the potential to reduce the cost of phenotyping and breeding cycles (Meuwissen et al., 2001) can help increase the rate of genetic gain especially in the case of quantitative traits. In the first step, genomic estimated breeding values (GEBVs) are estimated using a training set and different prediction models, and best prediction models are then used to select new germplasm developed by hybridization prior to field evaluation. The application of genomic prediction depends on the population size, marker density, model performance, heritability of the trait, training population size, and breeding population relatedness (Daetwyler et al., 2008; Bassi et al., 2016). In wheat, genomic prediction studies have been reported to predict rust resistance in diverse wheat landraces (Daetwyler et al., 2014; Crossa et al., 2016), landraces from Afghanistan (Tehseen et al., 2021), tetraploid wheat (Azizinia et al., 2020), and improved wheat germplasm (Ornella et al., 2012; Rutkoski et al., 2014; Bassi et al., 2016; Juliana et al., 2017).

This study was designed for: (i) evaluating the diversity for stripe rust resistance in 193 SYN-DERs against prevailing *Pst* races in Pakistan; (ii) conducting a GWAS analysis in SYN-DERs for resistance loci to the prevailing *Pst* races and identifying the linked SNP markers that could be deployed in marker-assisted selection (MAS); (iii) comparing genomic prediction accuracies for stripe rust resistance at seedling and adult plant stages using different models with two genotyping platforms, and (iv) determining whether some derivatives carry un-characterized genes for *Pst* resistance.

## Materials and Methods

### Plant Materials and Experimental Sites

A panel containing 193 SYN-DERs were evaluated in this study (Supplementary Table 1). The details of the germplasm have been described earlier (Afzal et al., 2019). Briefly, the SYN-DERs were developed by crossing elite cultivars and advanced lines of spring wheat with synthetic hexaploidy wheats in several combinations (refer to a pedigree for details of primary synthetic hexaploid wheat accessions numbers). The field experiments were conducted at the National Agricultural Research Centre (NARC), Islamabad (33° 0′N, 73° 4′E) and Cereal Crop Research Institute (CCRI), Nowshera (34° 1′N, 72° 2′E) Khyber Pakhtunkhwa, Pakistan, in the winter field seasons of 2015–2016 and 2016–2017.

### Seedling Stage Phenotyping

Seedling screening against stripe rust was performed at the Crop Disease Research Institute (CDRI), Murree, Pakistan under controlled conditions. Small plastic pots (8 cm × 10 cm) were filled with standard potting mix (soil and nursery substrate, 3:1), and were used to grow 5–6 plants of each accession including the susceptible wheat check cv. Morocco. The plants were grown in a glasshouse maintained at 50% humidity and 20°C. Genotypes were assessed for infection type responses to five *Pst* races: *Pst.*571242, *Pst.*571262, *Pst.*140202, *Pst.*571243, and *Pst.*173262 coded as Wang et al. (2016) and maintained at CDRI, Murree laboratory. These stripe rust races are frequently found in the yellow rust prone areas of Pakistan. The virulence and avirulence formulas for the isolates are provided in Table 1. The *Pst* isolates maintained at −80°C were heat shocked in a water bath at 42°C for 5 min. The mixture of petroleum ether (Merck Cat # 1.01775.2500) and paraffin oil (Merck Cat # 1.07162.1000) in a ratio of 4:1 was used to suspend the rust spores for inoculation on 10-day-old seedlings, at the two-leaf growth stage. The inoculum was applied using a fine mist atomizer. After inoculation, the mineral oil was allowed to evaporate, and the seedlings were then placed in a tray and watered. The *Pst* inoculated plant trays were shifted to a dark dew chamber at 100% relative humidity, 10°C temperature, and a light regime of 16 h light and 8 h dark for 24 h. Plants were then moved to a clean glasshouse under controlled temperature conditions of 15–18°C and 50% relative humidity. The same light/dark regime was continued during the rust evaluation. Water was non-limiting and recommended doses of liquid fertilizer were applied. Seedlings were treated with a growth inhibitor (Maleic Hydrazide) to slow plant development thus ensuring even disease infection and development. Notes on rust infection types were taken using a 0–9 scale (McNeal et al., 1971) on the 20th day of inoculation when susceptible genotype Morocco exhibited maximum infection. Seedling infection types (ITs) were classified as resistant with 0–4 (R), moderately resistant with score 5–6 (MR), and moderately to highly susceptible with score 7–9 (MS).

**TABLE 1:** Virulence profile of Pst races used in this study.

Pathotype	Virulence on genes	Avirulence on genes
*3.Pst.*140202	*Yr6, Yr7, Yr27*, and *YrExp2*	*Yr1, Yr5, Yr8, Yr9, Yr10, Yr15, Yr17, Yr24, Yr32, Yr43, Yr44, YrSp, YrTr1*, and *YrTye*
*5.Pst.*173262	*Yr6, Yr7, Yr8, Yr9, Yr15, Yr17, Yr27, Yr43, Yr44*, and *YrExp2*	*Yr1, Yr5, Yr10, Yr24, Yr32, YrSP, YrTr1*, and *YrTye*
*1.Pst*.571242	*Yr1, Yr6, Yr7, Yr8, Yr9, Yr17, Yr27, Yr43*, and *YrExp2*	*Yr5, Yr10, Yr15, Yr24, Yr32, Yr44, YrSP, YrTr1*, and *YrTye*
*4.Pst*.571243	*Yr1, Yr6, Yr7, Yr8, Yr9, Yr17, Yr43, YrExp2*, and *YrTye*	*Yr5, Yr10, Yr15, Yr24, Yr32, Yr44, YrSP*, and *YrTr1*
*2.Pst*.571262	*Yr1, Yr6, Yr7, Yr8, Yr9, Yr17, Yr27, Yr43, Yr44*, and *YrExp2*	*Yr5, Yr10, Yr15, Yr24, Yr32, YrSP, YrTr1*, and *YrTye*

### Adult Plant Disease Phenotyping

The diversity panel and a susceptible check (Morocco) were planted in 4 rows of 30 cm spacing and 2 m of length at NARC, Islamabad, and CCRI, Nowshera in 2015–2016 for screening for adult plant stripe rust resistance. The stripe rust susceptible cultivar Morocco was planted every 20th row to assist the spread of the rust epidemic. Inoculation was carried out using the *Pst* inoculum consisting of races used in this study. The inoculum was prepared by mixing rust spores mixture in liquefied petroleum ether (Merck Cat#1.01775.2500) and paraffin oil (Merck Cat # 1.07162.1000) in a ratio of 4:1 (V/V). The inoculum was sprayed with the help of a ULV sprayer on the rust spreader cultivar Morocco at the booting stage in both years at both field locations because this stage coincides with the favorable climatic conditions for rust spread. Rust infection and severity percentages were recorded when the genotype Morocco reached 70–80% severity. Rust scores were recorded three times each season at 1-week intervals to avoid disease escape. Wheat response to infection [infection types (IT)] was recorded using a 0–9 scale (Line and Qayoum, 1992). Yellow rust disease severity (DS) was noted as % infected leaf area of the host genotypes.

### Analyses of Variance, Heritability, and Correlation

Analyses of variance of yellow rust infection types and disease severity from adult plant field evaluation were done across years and environments using a linear mixed model to test for additive variance between genotypes, environments, and the interactions between genotypes by environments. In the mixed linear model, genotypes, and environments were used as fixed and years as random factors. Broad-sense heritability (H^2^) was calculated using an ANOVA model to estimate variance components on a genotype mean basis.


$$H^{2}=\frac{\sigma_{g}^{2}}{\sigma_{g}^{2}+\frac{\sigma_{ge}^{2}}{y}+\frac{\sigma_{e}^{2}}{yr}}$$

where, $\sigma_{g}^{2}$ is the genotypic variance, $\sigma_{e}^{2}$ is the environment variance, $\sigma_{ge}^{2}$σ *^2^_*gxe*_* is the genotype by environment interaction variance, and $\sigma_{e}^{2}$ error is the residual error variance, *y* is the number of years, and *r* is the number of replications within each experimental site. Pearson correlation coefficients (*r*) among experimental sites and cropping seasons were estimated to examine the consistency of infection types and disease severity across the environments. Statistical analyses of the present study were performed using R Statistical Software.

### DNA Extraction, and SNP Marker Genotyping

For genomic DNA extraction, five seeds of each SYN-DER accession were grown in 7 cm diameter disposable pots in a growth room. After 16–18 days of growth fresh leaf samples were taken to perform DNA extraction (Dreisigacker et al., 2013). Aliquant part of 50 μl DNA (50–100 ng/μl) for each sample was shipped in a 96-well plate arrangement for genotyping with high-density SNP markers, using the Wheat 90K SNP array (Wang et al., 2014), at the Department of Primary Industries, Victoria, Australia. The KASP marker for *Lr34/Yr18* was used to identify the durable rust resistance gene in SYN-DERs (Rasheed et al., 2016).

### Genome-Wide Association Analyses Using SNPs and Haplotypes

The GWAS for stripe rust responses recorded in seedling and field experiments was performed by the multi-locus GWAS methods. The population structure was inferred from the principal component analysis (PCA), and PC scores from the first five principal components were used as a Q matrix. The kinship matrix (K) was calculated from the TASSEL version 5.0. Quantitative trait nucleotides (QTNs) were identified by meMLM (Wang et al., 2016) and FASTmrMLM (Tamba and Zhang, 2018) methods, which are included in the R-package mrMLM v 3.1.^1^ For each trait, *P*-values were extracted from the TASSEL results. Manhattan and quantile-quantile (QQ; observed *P* values plotted against expected *P* values) were plotted using R package qqman (Turner, 2014).

The SNP linkage disequilibrium blocks (SNPLDBs) were constructed to identify the multiple alleles to fit the property of multiple alleles per locus in the SYN-DERs. The SNPLDB was constructed using RTM-GWAS software v1.2, which is publicly available at https://github.com/njau-sri/rtm-gwas (He et al., 2017). The output vcf from RTM-GWAS was used as a marker dataset for association analysis in TASSEL version 5.0.

### Genomic Prediction Models

This study used 11 marker-based prediction models to assess predictability (i.e., the correlation between predicted and observed trait values) of unobserved phenotypes. All prediction models differed from each other with respect to assumptions regarding estimation of markers effects. Among the parametric models included, an MLM-based prediction model, a genomic best linear unbiased prediction (GBLUP), was computed using the “BGLR” function of R package Bayesian generalized linear regression (BGLR) version 1.0.8 (Pérez and De, 2014). The GBLUP prediction model utilizes a realized genomic relation matrix (G) to model correlation among individuals (Vanraden, 2008; Habier et al., 2013). In addition, the Bayesian linear prediction models, i.e., Bayesian Ridge Regression (BRR), Bayesian least absolute shrinkage, and selection operator (BL), BayesA, BayesB, BayesC, and reproducing kernel Hilbert spaces regression (RKHS) were also used in prediction analysis. These Bayesian prediction models were also used with the function “BGLR” in the R package “BGLR.” For the ridge regression BLUP (rrBLUP) model, we used the “mixed.solve” function from rrBLUP R package version 4.6 (Endelman, 2011). To implement the elastic net (EN), we used the “glmnet” function implemented in the glmnet R package version 2.0–18 (Friedman et al., 2010). To compute EN, the value of alpha.5 was used. The EN model is a combination of ridge regression and LASSO. In addition, non-parametric models, i.e., relevance vector machines (RVM) and Gaussian Processes (GP), were used to build a GS prediction model. The “rmv” and “gausspr” functions from kernlab R package version 0.9–27 (Karatzoglou et al., 2007) were used for RVM and GP modeling. To verify the predictability of the 11 models in the SYN-DER population, we evaluated the prediction accuracy by 10-fold cross-validation using a training set randomly apportioned into each fold. The data were partitioned into training population (90%) and validation population (10%) sets.

## Results

### Phenotypic Variations for Stripe Rust Resistance in the SYN_DER Population

The response of the 193 SYN-DERs and check cultivars to *Pst* was assessed in four environments (two locations × 2 years) under high disease pressure. ANOVA showed highly significant (*P* < 0.001 and *P* < 0.01) differences among genotypes both for specific locations (Islamabad and Nowshera) and across locations (combined data) (Table 2). The variance components for environments were significant (*P* < 0.01 and *P* < 0.05). Similarly, genotypes by environment interactions for IT were significant (*P* < 0.05) at Nowshera and across locations buts non-significant for ISB.

**TABLE 2:** Mean response to *Puccinia striiformis* f. sp. tritici infection, estimates of variance components, and heritability.

Parameters	Islamabad (ISB)		Nowshera (NWS)		Across Locations	
	IT (0–9)	Severity (%)	IT (0–9)	Severity (%)	IT (0–9)	Severity (%)
Minimum	0.0	0.0	0.0	0.0	0.0	0.0
Mean	3.1	15.7	2.8	11.8	2.9	13.8
Maximum	8.5	90	8.5	80	8.5	85
σ^2^g	2.9***	278.6***	4.9***	247.7***	3.7***	255.8**
σ^2^e	0.9**	127.3*	1.9**	114.7**	1.5**	129.6**
σ^2^ge	3.8^ns^	406.0^ns^	6.8*	362.5^ns^	5.3*	385.4^ns^
σ^2^_*e*_	1.92	1.80	1.80	1.92	1.92	1.92
Heritability	0.75	0.68	0.71	0.68	0.72	0.66

σ^2^g, estimate of genotypic variance; σ^2^e, estimate of environmental variance; σ^2^ge, estimate of genotype x environment variance; σ^2^_e_, estimate of residual variance; H2, heritability; IT, infection type; DS, disease severity; ns, not significant; *P < 0.05; **P < 0.01; and ***P < 0.001.

**FIGURE 1:**
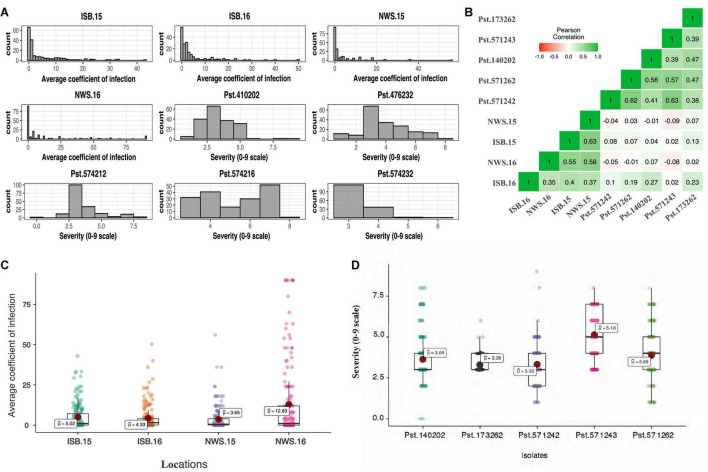
Histogram showing frequency distribution for the average coefficient of infection (ACI) at four locations, *viz.* Islamabad-2015 (ISB.15), Islamabad-2016 (ISB.16), Nowshera-2015 (NWS.15), and Nowshera-2016 (NWS.16), and disease severity (0–9 scale) against five Pst isolates **(A)**, boxplots for ACI at four locations **(B)**, and disease severity against five Pst isolates **(C)**, and coefficient of correlation across isolates and locations **(D)**.

The frequency distribution of ITs displayed by the SYN_DERs in response to the five *Pst* races is presented in Figures 1A,B. Of the lines tested, 78% (152), 63% (122), 79% (153), 38% (75), and 80% (156) of accessions showed seedling resistance to *Pst.571242*, *Pst.571262, Pst.140202, Pst.571243*, and *Pst.173262*, respectively. Notably, 18 (9%) of the genotypes showed seedling resistant infection types to all five *Pst* races.

The population showed a wide range of ITs across the environments. At Nowshera, 12% of accessions (24 genotypes) were highly resistant, 21% (41 genotypes) were showed resistant reactions, and 3 genotypes (1.5%) were highly susceptible. At ISB, 3% (6 genotypes) were highly resistant, 12% (24 genotypes) were resistant, and 1% (2 accessions) showed highly susceptible reactions (Figure 1). Eighteen (9%) of accessions were resistant in both cropping seasons at both experimental locations. Broad sense heritability (*H*^2^) for IT and disease severity ranged from 0.66 to 0.75% (Table 2).

Pearson correlation coefficients between stripe rust IT and disease severity between Islamabad and Nowshera in both years are presented in Figure 1E. Correlations were 0.51 and 0.59 for ITs, and 0.38 and 0.61 for disease severity at Islamabad and Nowshera, respectively. The correlations between Islamabad and Nowshera for ITs in 2015 and 2016 were 0.65 and 0.40, respectively. The respective disease severity correlations were 0.63 and 0.30. All five *Pst* races evaluated for ITs were significantly and positively correlated to each other and values ranged from 0.34 to 0.61 (Figure 1D). Seedling infection types and disease severity of *Pst.*140202 and *Pst.173262* were positively and were significantly correlated with adult plant ITs and disease severity in Islamabad in 2016.

### SNP and Haplotype Variations in the Synthetic-Derived Diversity Panel

**FIGURE 2:**
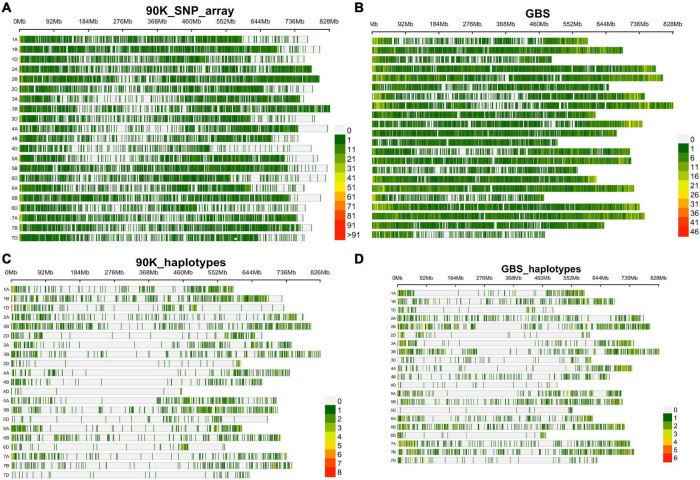
SNP density and distribution in all 21 wheat chromosomes using 90K SNP array and GBS characterized in SYN-DERs, **(A)** in 90K SNP array, **(B)** in GBS, **(C)** haplotype density using 90K SNP array, and **(D)** haplotype density using GBS platform.

Two genotyping platforms, 90K SNP array and GBS, were used for GWAS. For the 90K SNP array, 29,632 SNP markers were retained after removing SNPs with missing data of >10% and minor allele frequency of <0.05. Figure 2A shows SNP marker density on each wheat chromosome. For GBS, out of 236,327 SNPs identified, 47,122 were finally used after removing SNPs with >10% missing data, and <5% minor allele frequency (Figure 2B).

Haplotype blocks were constructed using both genotyping platforms using the block partitioning approach with CIs based on genome-wide LD (D^/^) patterns (Gabriel et al., 2002), and implemented in RTM-GWAS (He et al., 2017). Table 3 describes the number of haplotype blocks, the range and average size of blocks in terms of kb, and the range and average number of SNPs comprising each haplotype block on each chromosome. In the 90K SNP array, 19,070 LD blocks were constructed (Figure 2C), out of which 3,325 blocks contained more than two haplotype (alleles) (Table 3). The maximum number of haplotypes (*n* = 304) were constructed on chr2B, while the minimum was on chr4D (*n* = 10). On an average, the haplotype block size ranged from 5.5 Mb (chr7D) to 11.5 Mb (chr3D). The number of SNPs in each haplotype block was minimum 2 and maximum 14. In GBS, the number of blocks ranged from 16 (chr5D) to 364 (chr7B) (Figure 2D). The haplotype block size ranged from 2 to 19.6 Mb (chr3B), while SNPs/block ranged from 2 to 11 (chr3B and chr6A).

**TABLE 3:** Haplotype blocks on wheat chromosomes, their number, block size, and number of SNPs per block using 90K SNP array and genotyping-by-sequencing (GBS) platform.

	90K SNP array					GBS				
	*N*	Block size (kb)		SNPs		N	Block size (kb)		SNPs	
Chr		Range	Mean	Range	Mean		Range	Mean	Range	Mean
1A	203	3–99871	10339	2–8	2.76	177	2–19011	99093	2–8	2.76
1B	302	8–98626	7957	2–11	3.03	232	2–17887	98552	2–7	2.68
1D	137	9–85350	7191	2–8	2.69	52	3–16674	98478	2–5	2.65
2A	205	8–96300	7101	2–9	2.79	238	2–15837	99436	2–8	2.6
2B	304	2–99707	8066	2–14	3.06	335	2–18030	99593	2–14	2.8
2D	120	9–99556	6590	2–8	2.72	54	18–16323	97876	2–5	2.41
3A	157	12–95781	9254	2–8	3.05	185	2–18778	99440	2–8	2.84
3B	204	4–95726	8039	2–12	3.14	342	2–19638	99474	2–11	2.76
3D	41	5–91287	11581	2–9	2.88	72	6–14033	97701	2–8	2.39
4A	136	11–78450	5730	2–14	2.84	181	2–20901	99366	2–8	2.91
4B	117	6–90285	7816	2–8	2.84	138	2–12206	98672	2–7	2.38
4D	10	13–91196	15167	2–5	2.8	23	3–11454	92585	2–6	2.43
5A	202	3–99690	8305	2–11	2.99	186	3–15977	97276	2–8	2.65
5B	283	8–95328	10391	2–13	3.2	271	2–20623	99815	2–7	2.82
5D	67	7–99753	10919	2–11	2.87	16	7–19401	91446	2–5	2.56
6A	179	4–92210	11380	2–10	3.02	190	2–14164	98516	2–11	2.65
6B	206	2–98128	6607	2–10	2.8	329	2–16501	99871	2–9	2.68
6D	62	10–93126	7480	2–6	2.68	46	3–15001	98599	2–6	2.52
7A	182	3–89043	8119	2–13	3.04	292	2–18888	99514	2–9	2.8
7B	169	6–95920	8702	2–19	3.24	364	2–17576	99805	2–10	2.64
7D	39	37–48011	5530	2–6	2.41	79	2–15770	99555	2–7	2.59

### Association Analysis for Seedling Resistance to *Puccnina striiformis* in Synthetic-Deriveds

In total, 23 QTNs were identified for seedling resistance against five races in the SYN-DERs populations (Table 4). Eight QTNs were associated with seedling resistance against Pst.571242, of these the QTN on chr5B at 580.6 Mb was identified by both 90K SNP array and GBS and accounted for 10.5% of the total phenotypic variation. Only two QTNs were identified for resistance against Pst.571262 on chr3D and 7A, and explained 8.5 and 6.9% of the total variation, respectively. Five QTNs were detected against Pst.140202: these explained 2.3 to 10.9% of the total variation and were distributed on chr2D, chr3A, chr3B, chr3D, and chr5B. Seven QTNs were identified for resistance against Pst.571243 and accounted for 7.9 to 15.3% of the total variation. Only one QTN was identified for resistance against Pst.173262 on chr7A and explained 7.6% of the total variation.

**TABLE 4:** Quantitative trait nucleotides (QTNs) associated with resistance to *Pst* races at seedling stage in SYN-DER panel using 90K and GBS markers.

Race	SNP	Alleles*^a^*	Chr	Pos	QTN effect*^b^*	LOD score	−log10(p)	*r*^2^ (%)*^c^*	MAF*^d^*
Pst.571243	IWB72742	G/A	1B	300.6	–0.74	4.58	5.36	15.39	0.21
Pst.571243	1B_338552631	T/G	1B	338.6	1.05	3.74	4.48	9.18	0.06
Pst.571243	IWB73197	T/G	2B	152.2	–0.61	3.72	4.45	10.76	0.24
Pst.140202	2D_82307885	G/A	2D	82.3	0.86	4.18	4.94	10.9	0.1
Pst.140202	3A_701489529	C/T	3A	701.5	–0.65	3.48	4.21	5.29	0.08
Pst.140202	3B_180646490	T/C	3B	180.6	–0.57	3.67	4.41	9.98	0.23
Pst.140202	IWB26725	G/A	3D	367.4	0.48	3.29	4.01	6.09	0.15
Pst.571262	IWB1577	T/C	3D	439.7	–0.45	5.01	5.81	8.53	0.5
Pst.571242	IWB24288	A/G	3D	447.1	0.4	4.57	5.35	8.95	0.39
Pst.571243	4A_659618327	T/C	4A	659.6	0.55	3.61	4.35	11.3	0.42
Pst.571242	4B_11905357	G/A	4B	11.9	0.48	6.88	7.74	11.93	0.3
Pst.571242	IWB5827	T/C	4B	603.1	–0.39	3.91	4.66	9.15	0.44
Pst.571243	4B_609362872	A/C	4B	609.4	–0.49	4.93	5.72	9.26	0.48
Pst.571242	5A_363980539	A/G	5A	364.0	–0.32	3.43	4.15	5.3	0.31
Pst.571242	5A_590355732	C/T	5A	590.4	–0.36	3.49	4.21	7.91	0.48
Pst.571242	IWB28556	A/G	5A	620.6	–0.37	3.61	4.35	7.13	0.31
Pst.140202	IWB27708	A/G	5B	2.3	–0.22	3.39	4.11	2.34	0.32
Pst.571242	IWA3089	C/T	5B	580.4	0.43	3.29	4.01	10.6	0.47
Pst.571242	5B_580647907	T/C	5B	580.6	–0.75	7.24	8.11	10.52	0.08
Pst.571243	5B_580647907	T/C	5B	580.6	–0.77	4.19	4.96	7.19	0.09
Pst.571243	IWB35933	C/T	5D	521.4	–0.62	3.4	4.12	8.94	0.22
Pst.173262	7A_529833812	G/C	7A	529.8	–0.2	3.06	3.76	7.6	0.16
Pst.571262	7A_696929784	G/T	7A	696.9	–0.84	3.44	4.17	6.95	0.06

*^a^Resistance allele is underlined.*

*^b^QTN effect is negative if minor allele is increasing phenotype and positive if major allele is increasing phenotype value.*

*^c^Phenotypic variation explained by the QTN.*

*^d^Minor allele frequency.*

### Association Analysis for Adult Plant Resistance to *Puccnina striiformis*

**FIGURE 3:**
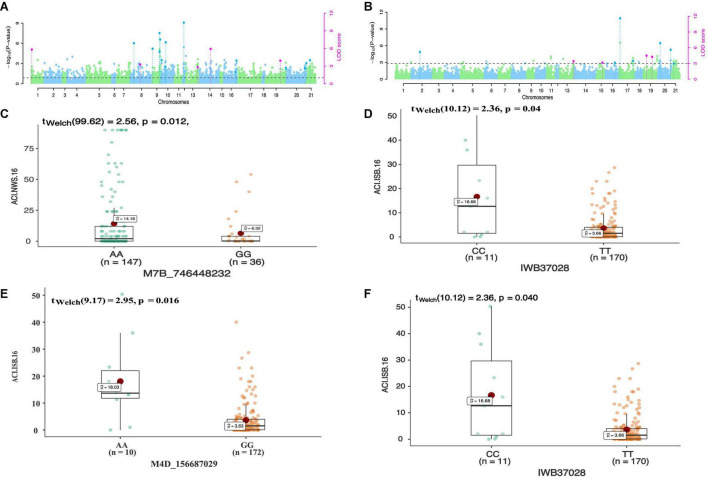
Manhattan plots showing distribution of *p*-value on –log(10) scale for SNPs associated with an average coefficient of infection (ACI) at Nowshera-2016 (NWS.16) using 90K SNP array **(A)** and GBS markers **(B)**. The allelic effects of SNPs on chr7B **(C)**, chr6B **(D)**, chr4D **(E)**, and chr6B **(F)** are shown as boxplots. Each boxplot shows the distribution of the average coefficient of infection (ACI) in a relevant environment for both allelic states of the SNP marker.

**FIGURE 4:**
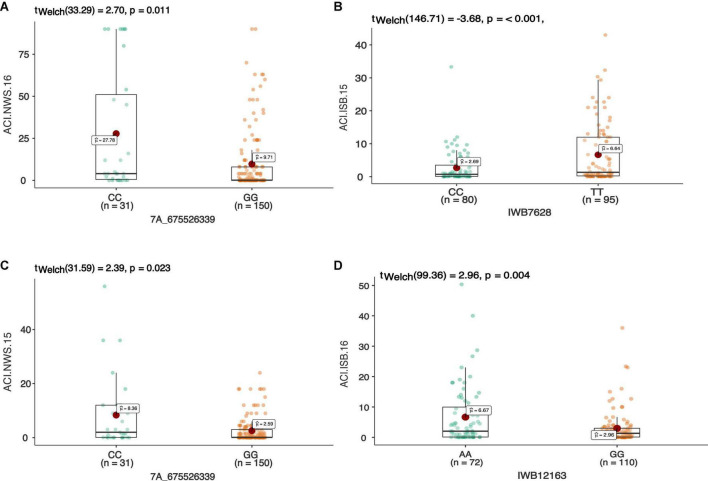
The allelic effect of SNP 7A_675526339 on chr7A associated with the average coefficient of infection at Nowshera (NWS) in both years 2015 and 2016 **(A,B)**. The allelic effects of IWB7628 on chr1A, and IWB12163 on chr7B on the ACI at Islamabad-2015 **(C)**, and Islamabad-2016 **(D)**, respectively. Each boxplot shows the distribution of the average coefficient of infection (ACI) in a relevant environment for both allelic states of the SNP marker.

**FIGURE 5:**
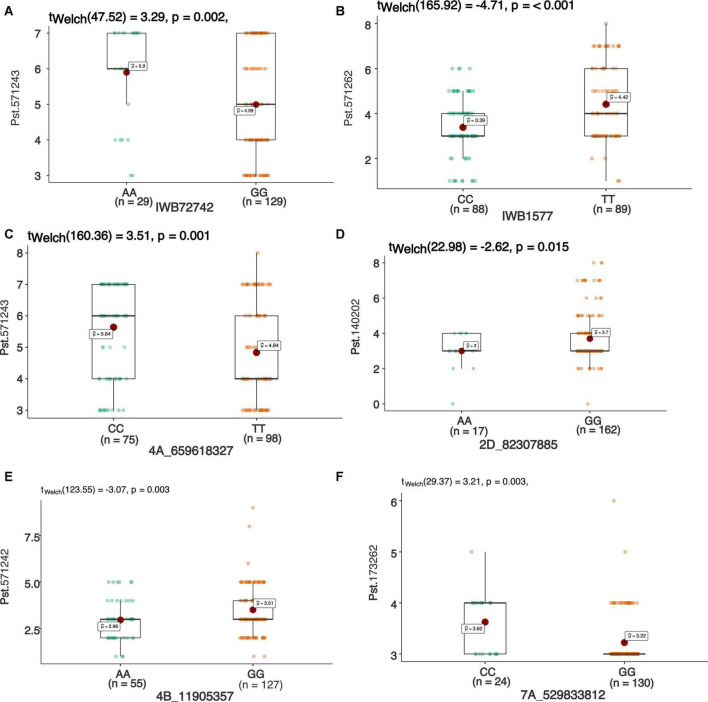
Box plots showing allelic effects of SNPs associated with resistance against stripe rust with highest phenotypic effect at seedling stage against race Pst.571242 **(A)**, Pst.571262 **(B)**, Pst.571243 **(C)**, Pst.140202 **(D)**, Pst.571243 **(E)**, and Pst.173262 **(F)**. Each boxplot shows the distribution of the average coefficient of infection (ACI) in a relevant environment for both allelic states of the SNP marker.

**FIGURE 6:**
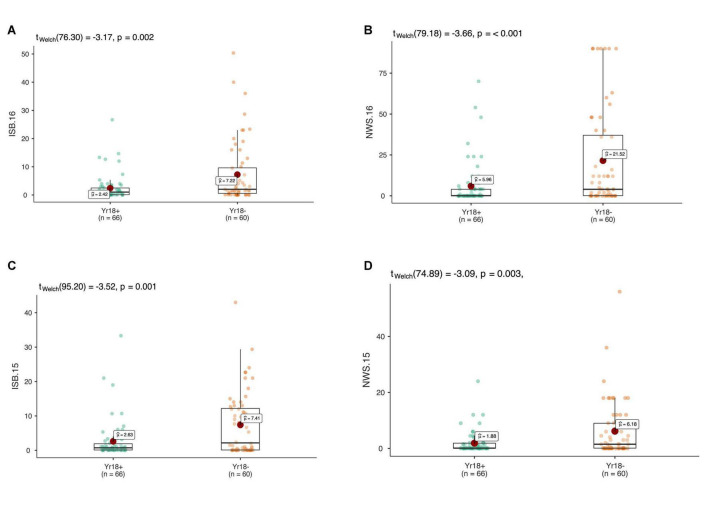
The allelic effects of the durable rust resistance gene *Lr34/Yr18* on the average coefficient of infection (ACI) in four environments at adult plant stage in ISB.15 **(A)**, ISB.16 **(B)**, NWS.15 **(C)**, and NWS.16 **(D)**. Each boxplot shows the distribution of the average coefficient of infection (ACI) in a relevant environment for both allelic states of the SNP marker.

In total 68 QTNs were identified for adult plant resistance against Pst in SYN-DERs (Table 5). Fourteen QTNs were identified for ISB.15, 19 for ISB.16, 18 for NWS.15, and 17 for NWS.2016. These QTNs were detected on all chromosomes except chr1D, chr2D, and chr4B. Figure 3 shows Manhattan plots for significant SNPs associated with resistance to Pst at NWS.16 using a 90K SNP array (Figure 3A) and GBS (Figure 3B). The allelic effects of associated SNPs are shown as box plots (Figures 3C–F). The phenotypic variation explained by the QTNs ranged from 3.2% (96.1 Mb at chr7D) to 29.3% (55.5 Mb at chr3B). Some QTNs were identified by both genotyping platforms, i.e., at 55.5 Mb on chr3B for resistance to *Pst* at ISB.15, and 560.4 Mb at chr1A for NWS.15. A QTN at 53.4–58.1 Mb on chr1A associated with NWS.15 and NWS.16, and another on chr7A at 675–676 Mb associated with NWS.15 and 16 (Figures 4A,B). Similarly, QTN on chr7B at 711–727 Mb was associated with resistance to Pst at NWS.16 and ISB.16 (Figure 4C). Some QTNs were associated with resistance to *Pst* at multiple environments including QTN at 3.1–3.9 Mb on chr1A associated with ISB.15, ISB.16, and NWS.15 (Figures 4C,D). Interestingly some QTNs were associated with both seedling and adult plant resistance, i.e., the QTN on chr1B at 300–327 Mb was associated with *Pst.571243* at ISB.16 (Figure 5A), and the QTN at 152–163 Mb on chr2B associated with Pst.571243 at NWS.16. Similarly, a QTN at 355–367 Mb on chr3D was associated with Pst.140202 at NWS.15 (Figure 5B). The QTNs of chr4A, chr2D, and chr4B were associated with Pst.571243, Pst.140202, and Pst.571242, respectively (Figures 5C–E). A QTN on chr7A at 675–696 Mb identified in NWS.15 and 16 was also associated with *Pst.571262* (Figure 5F). The allelic effects were also determined for the durable rust resistance gene *Yr18*, and the resistance allele was significantly associated with resistance to Pst in all four environments, i.e., ISB.16 (Figure 6A), NWS.16 (Figure 6B), ISB.15 (Figure 6C), and NWS.15 (Figure 6D).

**TABLE 5:** Quantitative trait nucleotides (QTNs) associated with resistance to *Pst* at adult plant stages in four environments in SYN-DER panel using 90K and GBS markers.

Environment	SNP	Alleles*^a^*	Chr	Pos (Mb)	QTN effect*^b^*	LOD score	−log10(p)	*r*^2^ (%)*^c^*	MAF*^d^*
ISB.15	IWB7628	T/C	1A	3.1	2.3	6.34	7.19	6.83	0.47
ISB.16	1A_3878168	G/T	1A	3.9	2.18	6.71	7.57	6.95	0.3
NWS.15	IWB4201	G/A	1A	4.0	–2.96	3.95	4.7	5.72	0.1
NWS.16	IWB21700	T/C	1A	534.3	–7.22	3.53	4.26	8.13	0.29
NWS.15	1A_560487941	G/A	1A	560.5	–2.35	4.52	5.29	5.98	0.17
NWS.15	IWB10188	G/A	1A	581.5	–3.08	5.11	5.91	9.09	0.15
ISB.15	1B_8591698	C/T	1B	8.6	–2.92	3.4	4.12	5.97	0.1
NWS.16	IWB64963	G/A	1B	86.8	7.81	5.69	6.51	11.1	0.48
ISB.15	IWB2120	A/C	1B	106.8	2.92	3.84	4.58	6.85	0.15
ISB.16	IWB49173	T/C	1B	327.8	3.11	5.19	6	10.57	0.19
ISB.15	1B_633336851	C/A	1B	633.3	2.65	3.74	4.48	3.26	0.09
ISB.15	1B_683306760	G/A	1B	683.3	–2.58	6.16	7	8.16	0.36
NWS.16	2A_566856454	G/T	2A	566.9	11.57	4.08	4.83	9.06	0.12
NWS.16	2B_163977776	G/A	2B	164.0	–9.38	5.72	6.55	8.33	0.18
NWS.16	2B_360129171	G/A	2B	360.1	–13.81	7.83	8.72	6.89	0.06
NWS.15	IWB35566	G/A	2B	783.2	–2.85	4.6	5.38	5.11	0.1
NWS.15	3A_130776756	C/T	3A	130.8	3.81	5.81	6.64	6.5	0.06
NWS.15	3A_503145562	A/G	3A	503.1	2.12	3.75	4.49	3.88	0.13
NWS.15	3A_736945971	A/T	3A	736.9	–2	5.46	6.28	7.22	0.41
ISB.15	IWA747	G/A	3B	55.5	–3.21	5.65	6.47	9.26	0.21
ISB.15	3B_55514953	T/C	3B	55.5	–6.08	14.26	15.27	29.31	0.18
NWS.16	3B_65339336	G/A	3B	65.3	–15.82	5.7	6.52	14.21	0.1
ISB.16	3B_470866042	A/G	3B	470.9	–2.83	4.72	5.51	4.71	0.09
ISB.16	3D_2620724	C/T	3D	2.6	–2.17	5.95	6.78	8.2	0.5
NWS.15	3D_355163225	T/C	3D	355.2	–1.94	3.36	4.08	3.69	0.16
ISB.16	3D_551073224	T/C	3D	551.1	–2.34	5.45	6.27	6.64	0.23
NWS.16	4A_438964494	C/T	4A	439.0	5.83	5.54	6.36	5.45	0.48
NWS.16	IWB68805	C/T	4A	733.6	4.91	3.35	4.07	3.75	0.3
ISB.16	4D_156687029	G/A	4D	156.7	–6.08	10.39	11.34	13.35	0.06
NWS.15	IWB33444	C/T	5A	481.9	–2.41	10.85	11.81	9.01	0.5
ISB.16	IWA4223	C/T	5A	670.4	–1.72	3.09	3.79	4.71	0.46
ISB.15	IWB7864	G/A	5B	2.6	–2.29	4.23	4.99	6.14	0.32
NWS.16	IWB65690	G/A	5B	10.8	8.23	6.46	7.31	12.44	0.49
NWS.15	IWB8592	G/A	5B	64.7	2.79	9.55	10.47	8.81	0.24
NWS.16	5B_207483057	G/A	5B	207.5	6.74	4	4.75	3.31	0.13
ISB.16	5B_471381890	A/G	5B	471.4	2.81	4.58	5.36	6.28	0.13
NWS.16	IWA2062	G/A	5B	542.6	–9.99	3.94	4.69	6.48	0.07
NWS.15	IWB65055	T/C	5B	692.6	–2.58	4.62	5.4	8.44	0.26
NWS.15	IWB14489	G/A	5D	133.5	–3.85	6.99	7.85	6.04	0.06
ISB.16	IWB9144	G/A	5D	487.6	–1.73	3.15	3.85	4.52	0.41
ISB.15	IWB30735	T/C	6A	297.7	–2.8	4.14	4.9	8.9	0.33
NWS.15	IWB66163	T/C	6A	415.9	–2.18	3.61	4.34	3.82	0.14
ISB.16	IWB40151	A/G	6A	546.6	–1.89	3.14	3.85	5.44	0.37
ISB.16	6A_595332866	T/C	6A	595.3	–2.64	3.94	4.69	4.1	0.09
ISB.16	IWB37028	T/C	6B	4.4	–5.97	11.61	12.58	14.01	0.06
NWS.16	6B_22858086	A/G	6B	22.9	–12.25	7.15	8.02	9.36	0.11
NWS.15	6B_31867138	C/T	6B	31.9	–4.97	9.13	10.05	10.2	0.06
ISB.15	6B_231490683	G/A	6B	231.5	2.46	4.29	5.06	5.98	0.25
NWS.16	6B_361469100	C/T	6B	361.5	–9.57	4.24	5.01	3.59	0.07
ISB.15	6B_419133836	G/A	6B	419.1	–3.17	6.3	7.14	6.62	0.15
NWS.15	6B_618067850	G/C	6B	618.1	–4.03	5.93	6.76	6.71	0.06
NWS.15	6D_436810635	G/A	6D	436.8	–3.16	5.47	6.29	10.85	0.17
ISB.16	IWB74161	C/T	7A	47.0	–1.79	3.34	4.06	5.05	0.43
NWS.16	7A_234640959	C/T	7A	234.6	5.9	4.71	5.49	5.49	0.43
ISB.16	IWB21762	C/T	7A	506.1	–2.21	4.53	5.3	6.44	0.28
ISB.16	7A_588284942	G/A	7A	588.3	1.78	3.86	4.61	4.34	0.3
NWS.16	7A_675526339	G/C	7A	675.5	–7.26	5.11	5.91	4.86	0.17
NWS.15	7A_676996750	G/A	7A	677.0	–3.29	5.28	6.08	7.02	0.09
ISB.16	IWB26214	C/T	7B	59.6	–3.19	6.87	7.73	8.24	0.14
NWS.15	IWA5939	T/C	7B	582.3	4.04	12.21	13.19	11.9	0.15
ISB.15	IWB13912	T/C	7B	692.6	–3.28	5.45	6.26	7.47	0.12
NWS.16	IWB48256	T/C	7B	711.5	–6.84	3.59	4.32	7.87	0.35
ISB.16	IWB12163	G/A	7B	727.5	–2.06	5.62	6.44	6.45	0.4
ISB.16	7B_746448232	A/G	7B	746.4	–1.87	3.49	4.22	3.87	0.2
NWS.16	IWB42068	A/G	7D	11.4	–5.84	3.69	4.43	6.05	0.4
ISB.15	IWB74163	A/C	7D	44.5	–3.07	9.65	10.58	12.08	0.41
ISB.15	IWB59266	A/G	7D	58.7	–3.11	4.95	5.74	7.59	0.14
ISB.16	7D_96173227	G/T	7D	96.2	1.82	4	4.75	3.25	0.17

*^a^Resistance allele is underlined.*

*^b^QTN effect is negative if the minor allele is increasing phenotype and positive if the major allele is increasing phenotype value.*

*^c^Phenotypic variation explained by the QTN.*

*^d^Minor allele frequency.*

### Haplotype Blocks Associated With Resistance to *Puccnina striiformis* at Seedling and Adult Plant Stages

**FIGURE 7:**
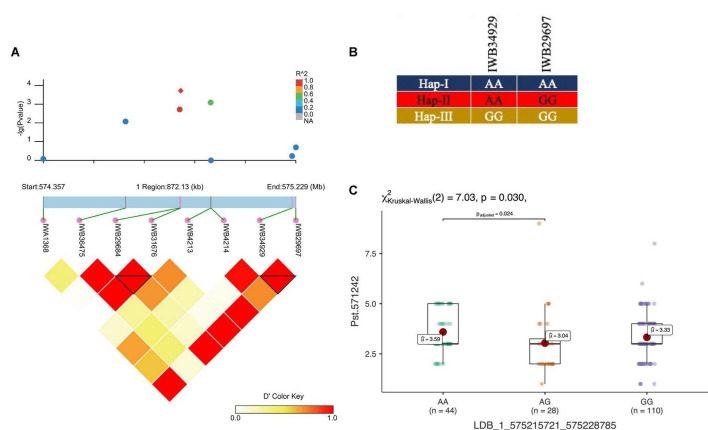
LD haplotype block with SNP positions **(A)**, variants of haplotype block **(B)**, and allelic effect of different haplotypes on resistance against Pst.571242 in the block for LBD_1_575215721_575228785 on chr1A **(C)**. Each boxplot shows the distribution of the average coefficient of infection (ACI) in a relevant environment for all allelic states of the SNP marker.

In total, three haplotype blocks were associated with seedling resistance against Pst.571242, Pst.140202, and Pst.173262 on chr1A, chr3B, and 7D, respectively (Table 6). The haplotype block on chr1A identified with the 90K SNP array was present at 575.2 Mb and contained six haplotypes, whose frequency ranged from 1.03 to 59% (Figures 7A,B). The effect of all three haplotypes of chr1A LD block is shown in Figure 7C. A haplotype block on chr3B by (GBS markers) was positioned at 125.8 Mb and had four haplotypes with a frequency of 67 to 1%. Similarly, the haplotype block on chr7D was present at 627.3Mb and contained five variants with a frequency between 1.5 to 79.2%. This haploblock is likely a homolog of the QTN identified on chr7A for resistance against the same race.

**TABLE 6:** Haplotypes associated with resistance to *Pst* at seedling and adult plant stages in SYN-DER wheats using 90K and GBS markers.

							Genotype (Frequency)	
	
Trait	Haplotype ID	Chr	Position	SNPs/ block	−log10(p)	Hap-I	Hap-II	Hap-III
ISB.16	LDB_1_25490035_25490120	1A	25490035	2	1.45E-06	GA (0.68)	AG (0.30)	GG (0.019)
**Pst.571242**	**LDB_1_575215721_575228785**	**1A**	**575215721**	**2**	**1.00E-08**	**GG (0.59)**	**AA (0.18)**	**AG (0.15)**
NWS.15	LDB_3_19112718_19129042	1D	19112718	2	1.19E-06	AA (0.74)	TG (0.25)	
ISB.15	LDB_3_488576303_488577792	1D	488576303	4	4.00E-07	ATGT (0.55)	GGAC:0.37	GGGT (0.02)
NWS.15	LDB_3_488576303_488577792	1D	488576303	4	1.00E-08	ATGT (0.55)	GGAC:0.37	GGGT (0.02)
ISB.15	LDB_4_30830742_30831056	2A	30830742	2	7.00E-08	CA (0.92)	TG:0.06	CG (0.01)
NWS.15	LDB_5_6258683_6338084	2B	6258683	9	3.00E-12	CATTCTTCA (0.43)	CACCCTTCA (0.18)	TGTTCCTCG (0.14)
Pst.140202	LDB_8_125880000_125930410	3B	125880000	4	6.91E-06	GACT (0.67)	GGTC (0.15)	AGTC (0.15)
NWS.16	LDB_10_111292188_111292941	4A	111292188	2	2.00E-09	GC (0.47)	AT (0.44)	GT (0.046)
**NWS.15**	**LDB_13_465541110_465541233**	**5A**	**465541110**	**2**	**5.00E-07**	**CG (0.58)**	**TA (0.35)**	**TG (0.02)**
**ISB.16**	**LDB_13_465541110_465541233**	**5A**	**465541110**	**2**	**1.00E-08**	**CG (0.58)**	**TA (0.35)**	**TG (0.02)**
**NWS.15**	**LDB_16_584678556_584680439**	**6A**	**584678556**	**2**	**8.00E-06**	**GT (0.60)**	**TG (0.31)**	**GG (0.025**
**NWS.16**	**LDB_17_15781175_15781777**	**6B**	**15781175**	**3**	**1.00E-07**	**CCG (0.34)**	**CTG (0.31)**	**TCT (0.25)**
Pst.173262	LDB_21_627325333_627325482	7D	627325333	2	6.00E-07	GC (0.79)	AT (0.103)	GT (0.06)

*Bold haplotype blocks are the loci also identified by SNP-GWAS. Only the top three most frequent haplotypes in each LD block are mentioned, and the values in parentheses are the frequencies of the relevant haplotypes in the diversity panel.

**FIGURE 8:**
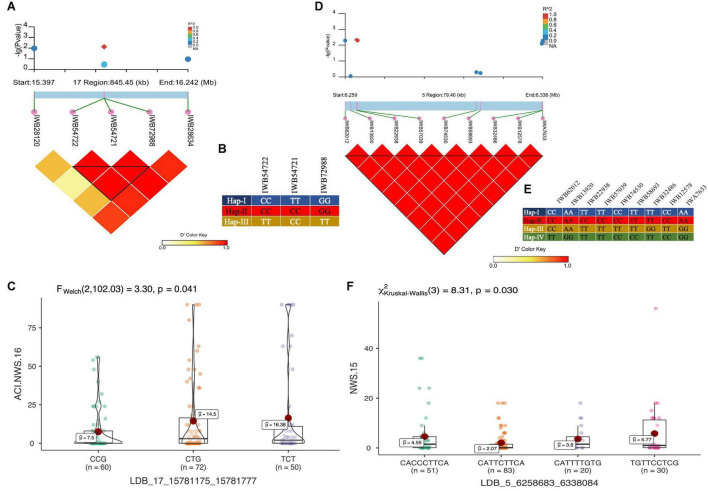
LD haplotype block with SNP positions, variants of haplotype block and allelic effect of different haplotypes in the block for LBD_17_15781175_15781777 on chr6B **(A–C)**, and LBD_5_6258683_6338084 on chr2B **(D–F)**. Each boxplot shows the distribution of the average coefficient of infection (ACI) in a relevant environment for all allelic states of the SNP marker.

In total, 11 haplotype blocks (two identified with GBS and nine with 90K SNP array) were associated with *Pst* resistance at the adult plant stage. Two haplotype blocks on chr5A and chr1A were associated with resistance to *Pst* at ISB.16, with three and six haplotype variants observed, respectively. The haplotype block on chr6B was associated with resistance to Pst at NWS.16 and consisted of three haplotypes (Figures 8A,B), where the Hap-II (CCG) significantly reduce the ACI (Figure 8C). Similarly, haplotype block on chr1A consisted of four haplotypes (Figures 8D,E), and Hap-II (CATTCTTCA) was associated with resistance to Pst at NWS.15 (Figure 8F). A haplotype block at 488 Mb on chr1D was associated with resistance to *Pst* at ISB.15 and NWS.15, while another haplotype block on chr5A at 465 Mb was associated with *Pst* resistance at ISB.16 and NWS.15. This haplotype block is likely the QTN at 481 Mb which was associated with APR at NWS.15. Five haplotype blocks were associated with *Pst* resistance at NWS.15 and these were distributed across chr1D (2), chr2B, chr5A, and chr6A. For the haplotype block on chr2B (6.2 Mb), 13 different haplotype variants were identified with a frequency ranging between 1.03 and 43%.

### Genomic Prediction for Resistance Against *Puccnina striiformis*

**FIGURE 9:**
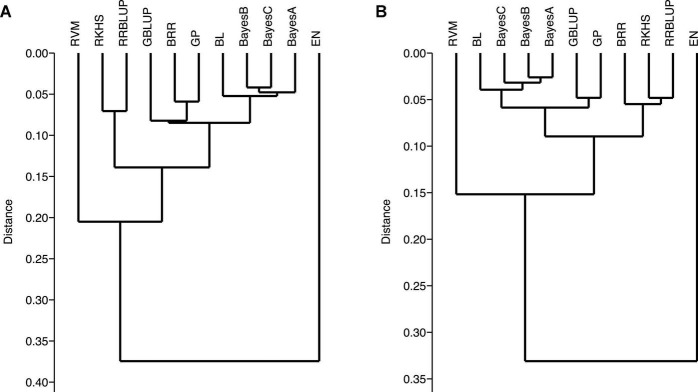
Ward’s hierarchical clustering on the prediction genomic values derived from the stripe rust infection types using 90K **(A)** and GBS **(B)** marker platforms. Genomic prediction models: BayesA, BayesB, BayesC, Bayesian ridge regression (BRR), Bayesian least absolute shrinkage and selector operator (BL), genomic best linear unbiased prediction (GBLUP), reproducing kernel Hilbert spaces regression (RKHS), elastic net (EN), relevance vector machine (RVM), Gaussian processor (GP), and ridge regression best linear unbiased prediction (rrBLUP).

Genomic-prediction analysis was conducted using a fivefold validation for *Pst* resistance at four locations and five *Pst* races using 11 different prediction models (Table 7). In the case of APR, prediction accuracies ranged from 0.23 (RKHS for ISB.15) to 0.511 (BL for NWS.15) using 90K markers, while prediction accuracies were relatively lower for GBS. Among the prediction models, BRR, BL, and GBLUP showed higher prediction accuracies compared to other models. Prediction accuracies were low for Pst.173262 and Pst.140202 using both GBS and 90K markers. The hierarchical clustering was used to classify the prediction models, which indicated that EN-based prediction accuracies were quite different than other models both for 90K and GBS markers (Figures 9A,B). Based on the 90K platform, all Bayes model (A, B, and C) and BL were quite similar in the prediction of reaction against Pst. BRR, GP, and GBLUP were quite similar in the case of the GBS platform (Figure 9A), while BRR was a bit different compared to GP and GBLUP in the case of 90K markers (Figure 9B).

**TABLE 7:** Genomic prediction accuracy using 11 different models for stripe rust resistance at four locations, and against five isolates at seedling stage using 90K SNP array and genotyping-by-sequencing (GBS) platform.

Markers		NWS.15	ISB.15	NWS.16	ISB.16	Pst.571242	Pst.571262	Pst.140202	Pst.571243
90K	BayesA	0.506 (0.052)	0.405 (0.053)	0.482 (0.07)	0.506 (0.038)	0.279 (0.113)	0.219 (0.074)	0.11 (0.05)	0.426 (0.059)
	BayesB	0.489 (0.055)	0.407 (0.05)	0.486 (0.07)	0.51 (0.044)	0.286 (0.111)	0.228 (0.079)	0.101 (0.051)	0.414 (0.062)
	BayesC	0.491 (0.061)	0.413 (0.052)	0.477 (0.077)	0.5 (0.044)	0.29 (0.104)	0.236 (0.076)	0.107 (0.055)	0.434 (0.061)
	BRR	0.488 (0.055)	0.395 (0.056)	0.502 (0.061)	0.498 (0.044)	0.264 (0.102)	0.236 (0.079)	0.139 (0.041)	0.413 (0.06)
	BL	0.511 (0.053)	0.392 (0.047)	0.47 (0.078)	0.493 (0.043)	0.275 (0.101)	0.24 (0.073)	0.102 (0.05)	0.42 (0.059)
	GBLUP	0.468 (0.058)	0.393 (0.048)	0.472 (0.069)	0.494 (0.044)	0.259 (0.103)	0.241 (0.077)	0.106 (0.038)	0.396 (0.059)
	RKHS	0.354 (0.054)	0.235 (0.054)	0.39 (0.081)	0.386 (0.062)	0.205 (0.094)	0.221 (0.064)	0.061 (0.049)	0.226 (0.084)
	EN	0.48 (0.062)	0.413 (0.06)	0.482 (0.069)	0.491 (0.043)	0.227 (0.104)	0.214 (0.085)	0.117 (0.033)	0.402 (0.056)
	RVM	0.505 (0.067)	0.396 (0.072)	0.486 (0.069)	0.466 (0.049)	0.245 (0.108)	0.212 (0.06)	0.131 (0.043)	0.34 (0.054)
	GP	0.476 (0.058)	0.392 (0.06)	0.5 (0.058)	0.498 (0.048)	0.258 (0.107)	0.25 (0.076)	0.088 (0.038)	0.408 (0.057)
	RRBLUP	0.481 (0.057)	0.406 (0.052)	0.486 (0.069)	0.501 (0.042)	0.228 (0.099)	0.222 (0.078)	0.139 (0.035)	0.405 (0.058)
GBS	BayesA	0.449 (0.108)	0.442 (0.077)	0.391 (0.061)	0.399 (0.089)	0.168 (0.047)	0.102 (0.07)	0.079 (0.053)	0.23 (0.051)
	BayesB	0.421 (0.118)	0.432 (0.083)	0.386 (0.06)	0.405 (0.086)	0.146 (0.046)	0.117 (0.067)	0.067 (0.056)	0.227 (0.048)
	BayesC	0.421 (0.114)	0.412 (0.084)	0.395 (0.061)	0.396 (0.084)	0.157 (0.053)	0.122 (0.07)	0.097 (0.053)	0.236 (0.044)
	BRR	0.407 (0.114)	0.426 (0.079)	0.398 (0.061)	0.428 (0.084)	0.146 (0.05)	0.106 (0.067)	0.062 (0.059)	0.229 (0.048)
	BL	0.428 (0.111)	0.399 (0.087)	0.38 (0.062)	0.391 (0.093)	0.15 (0.05)	0.109 (0.071)	0.054 (0.056)	0.241 (0.048)
	GBLUP	0.417 (0.111)	0.435 (0.081)	0.366 (0.069)	0.388 (0.086)	0.151 (0.051)	0.117 (0.069)	0.013 (0.054)	0.225 (0.05)
	RKHS	0.403 (0.115)	0.424 (0.082)	0.36 (0.064)	0.407 (0.09)	0.104 (0.043)	0.107 (0.07)	0.039 (0.052)	0.228 (0.052)
	EN	0.26 (0.086)	0.33 (0.109)	0.17 (0.104)	0.379 (0.066)	0.256 (0.049)	0.002 (0.087)	−0.01(0.07)	0.084 (0.065)
	RVM	0.486 (0.106)	0.381 (0.08)	0.346 (0.072)	0.439 (0.084)	0.06 (0.04)	0.038 (0.071)	0.171 (0.06)	0.224 (0.083)
	GP	0.413 (0.114)	0.427 (0.087)	0.372 (0.069)	0.466 (0.084)	0.139 (0.049)	0.133 (0.071)	0.048 (0.066)	0.246 (0.056)
	RRBLUP	0.398 (0.109)	0.421 (0.082)	0.388 (0.061)	0.385 (0.087)	0.079 (0.056)	0.104 (0.07)	0.001 (0.053)	0.213 (0.055)

*Genomic prediction models: BayesA, BayesB, and BayesC. BRR, Bayesian ridge regression; BL, Bayesian least absolute shrinkage and selector operator; GBLUP, genomic best linear unbiased prediction; RKHS, reproducing kernel Hilbert spaces regression; EN, elastic net; RVM, relevance vector machine; GP, Gaussian processor; rrBLUP, ridge regression best linear unbiased prediction. The values in the parentheses are SDs of the prediction accuracies.*

## Discussion

### Stripe Rust Resistance in Synthetic-Deriveds at Seedling and Adult Plant Stages

The deployment of new, effective, and durable sources of resistance against *Pst* is required to reduce the risk of epidemics. Seven SYN-DERs were found to possess a high level of resistance against three *Pst* races, while six were resistant against all five races. It is likely that these SYN-DERs (SD37, SD38, SD73, SD85, SD104, SD172, and SD173) carry major stripe rust resistance genes. All five races used in the evaluation were avirulent to *Yr24/Yr26*, which was identified in synthetic hexaploid wheats and has been deployed in China and elsewhere (McIntosh et al., 2018). Most *Pst* races are avirulent to the *Yr24/Yr26* gene, however, races virulent to *Yr10* were also virulent to *Yr24/Yr26*, e.g., Australian *Pst* race 150 E16A + and Chinese *Pst* races V26-CH42, and V26-Gui22 (McIntosh et al., 2018). Since SYN-DER wheats are not extensively deployed in Pakistan and the races used in this study were the most virulent available races, it is likely that virulence to *Yr24/Yr26* is not common in the pathogen population in Pakistan. Therefore, the eight SYN-DERs could be an excellent source of resistance against *Pst* in Pakistan and other countries where virulence to this gene combination is not present.

At the adult plant stage, more than 110 SYN-DERs showed moderate to resistant responses against *Pst.* The field screening was carried out in ‘hot-spot’ areas of *Pst* incidence, thus, this APR in SYN-DER could be usefully deployed against *Pst* races in the region. These results are in accordance with previous findings that APR occurs at a high frequency in synthetic hexaploid wheat (Zegeye et al., 2014; Bhatta et al., 2019). This is partly attributable to the fact that the A and B genomes of durum wheat are present completely in synthetic hexaploid wheat and partially in SYN-DERs. Previous studies indicate that *Pst* isolates from bread wheat are often avirulent on durum wheat (Aoun et al., 2021). Among the APR SYN-DERs, 68 carried the *Lr34/Yr18* gene, which is known to provide a partial resistance against all *Pst* races. The results also suggested the presence of *Lr34/Yr18* reduced overall incidence of *Pst* in all four environments against all five isolates. However, none of the SYN-DERs carried *Lr67/Yr46*, which was expected because this gene evolved after polyploidization and is mostly present in landraces from Pakistan and India (Riaz et al., 2016), while synthetic hexaploid wheats and parents used in SYN-DER did not have any introgression from Pakistan or Indian landraces.

### Quantitative Trait Nucleotides and Haplotypes Associated With *Puccnina striiformis* Resistance in Synthetic-Deriveds

Both platforms, i.e., GBS and 90K SNP array, effectively identified the loci associated with resistance to Pst, and some QTNs were common to both platforms. We have collected information for stripe rust resistance loci from 35 different studies (Supplementary Table 2) and compared our QTNs with previous findings. Among the *Yr* resistance genes, *Yr24/Yr26* is derived from synthetic wheats and widely deployed in synthetic wheat-based commercial cultivars in China (Zeng et al., 2014). Previously, GWAS identified several *Yr* resistance loci co-localized with known *Yr* genes including *Yr24/Yr26/Yr28* on chromosome 1B, *Yr48* on chromosome 5AL, *Yr32* on chromosome 2A, and *Yr19* on chromosome 5BL (Zegeye et al., 2014). Apart from *Yr24/Yr26*, it was expected that several of the SYN-DERs could carry *Yr32* because all five races are avirulent to this gene. One QTN and one haplotype were associated with resistance to *Pst* on chr2A at 566 and 30.8 Mb, respectively. The QTN at ∼566 Mb was likely to be *Yr32*; previously, the SNP AX-108752496 (similar position) was reported to be associated with *Pst* resistance (Wu et al., 2021). However, the minor allele provided resistance and its frequency at this QTN was 12% (*n* = 23), fourteen out of 23 SYN-DERs also possessed the durable rust resistance gene *Lr34/Yr18*. Therefore, these 14 accessions could carry both major and minor genes, thus, provide valuable donor sources for breeding programs.

Among the 32 seedlings and 68 APR QTNs, 18 had a phenotypic effect exceeding 10%. The largest effect QTN on chr3B at ∼55.4 Mb explained 29.3% of the variation was identified by both platforms in two environments. However, the phenotypic variation explained was relatively lower in ISB.16 (14.2%). Yao et al. (2020) previously identified a QTN at a similar position in Chinese wheat landraces (designated *QYr.nafu.3BS)*. Since the major allele provided resistance at this QTN, it is likely that this locus was responsible for the high frequency of the resistant SYN-DERs.

Previous studies identified QTNs for resistance to *Pst* on chromosomes 2A, 3B, 6A, and 7B in an association mapping panel of 181 SHWs (Zegeye et al., 2014). The QTNs and haplotypes identified on the D genome showed the potential of SYN-DERs for improving the *Pst* resistance in modern wheat cultivars. The same loci associated with seedling and APR to *Pst* on chr1B (∼300 Mb), chrr7A (∼506 Mb), chr2B (∼150 Mb), chr3A (∼701 Mb), chr3D (∼355 Mb), and chr5B (∼2.2 Mb). These loci could be used to discover potentially novel alleles of major stripe rust resistance genes. The genes *Yr18, Yr29, Yr30*, and *Yr78* have been widely used in wheat breeding (Wu et al., 2021). However, in our study, no SNP association was found in the vicinity of *Yr78* and *Yr30*. The QTN on chr1B at 683 Mb was likely to be *Yr29*, and a QTL *QYr.nwafu-1BL* was also identified in close proximity (Wu et al., 2021). The QTN on chr3A was identified as effective against Pst.140202, and a major gene *Yr75* is located nearby at ∼675 Mb, while the stem rust resistance gene *Sr15* was identified at the same position (Babiker et al., 2015). However, several loci identified in this study could not be compared with the previous studies due to the absence of a meta-analysis of stripe rust resistance loci in wheat. The establishment of such a framework would greatly enhance the validation and identification of loci associated with stripe rust resistance, particularly in GWAS studies.

Our study applied an LD based haplotype approach to discover loci associated with resistance to *Pst*. Until now, only one haplotype-based GWAS for stripe rust resistance has been reported (Wu et al., 2021). Previously, we used a haplotype-GWAS approach in SYN-DERs to identify the loci associated with drought adaptability (Afzal et al., 2019). The results confirmed that haplotype-GWAS was an effective strategy to increase the power of GWAS experiments. Here, we showed that haplotype-GWAS identified 9 out of 13 trait-associated loci where individual SNPs were ineffective. This was because haplotypes containing a group of closely linked SNP markers can increase the level of polymorphisms and overcome the limitation of using single SNP markers by creating more combinations (haplotypes). Several haplotypes associated with phenotypes in our study were not identified by SNP-GWAS and this could be due to many factors, including patterns of LD in the population, marker density, and the genetic architecture of the trait. The haplotype on chr6B (Figures 7A–C) associated with ARP was also identified by SNP-GWAS and is likely to be a new locus. Similarly, a haplotype consisting of nine SNPs on chr2B at 6.2 Mb (Figures 7D–F) was not identified by SNP-GWAS, and Hap-II, which provided a high level of APR was present in 83 accessions. Several genes and QTL have been identified on chr2B including *Yr32, Yr43, Yr44, Yr53, Yr72, Qyr.cim.2BS2,3*, and many more (Supplementary Table 3). However, none of these genes or QTL were located at the position of the haploblock as identified in this study, thus, it could be a new locus. Another haplotype on chr1A was in the proximity of QTL Qyr.nwafu-1AL at ∼587 Mb, which was previously reported using 90K and 660K markers. However, no major gene has been identified in this region. In conclusion, haplotype-GWAS proved to be a useful approach in combination with SNP-GWAS to improve the discovery of resistance loci.

### Genomic Prediction for Stripe Rust Resistance

The transition from phenotypic selection to marker-assisted selection, and now genome-wide selection, will allow breeders to improve the selection decision during the early filial generations. However, the success of genomic selection depends on several factors such as the heritability of the trait, phenotypic variation explained by markers, and appropriate genomic prediction models (Ali et al., 2020). Genomic prediction resulted in an accuracy of up to 85% for APR at ISB.16, although predictions at other locations were less accurate. Prediction accuracies were low to moderate for the three other environments using the 90K SNP array but higher for GBS markers. The reason for low prediction accuracies can be attributed to smaller population sizes and unrelated genotypes. Recently, the prediction accuracy for stripe rust resistance in wheat landraces from Afghanistan was observed to be between 0.33 to 0.38 (Tehseen et al., 2021). Among the prediction models used, GBLUP and BayesB were the most effective, while EN was the least. The results in this study supported previous genomic prediction studies, where GBLUP and similar models predicted the disease resistance more accurately than other models (Avni et al., 2017; Juliana et al., 2017; Tehseen et al., 2021).

## Conclusion

There is an ongoing need to identify new sources of resistance to *Pst.* The SYN-DERs provide valuable genetic resources for wheat improvement because they have high breeding value and are derived from primary synthetic hexaploidy wheats with D-genome contribution from *Ae. Tauschii.* Thus, SYN-DERs can be used to enhance the diversity of the D-genome in modern bread wheat but also the diversity of the A and B genomes because the synthetic wheats carry introgressions from durum wheat. More than 65 loci were identified in this study, which represent potentially important genes for race-specific and broad-spectrum resistance to stripe rust. Haplotype-GWAS should be a routine GWAS analytical approach to extend the discovery of genetic loci associated with phenotypes. The novel loci for resistance to stripe rust identified by SNP, and haplotype GWAS provide an arsenal of new alleles for resistance breeding. The SNP markers with large phenotypic effects for both all-stage resistance and APR can be converted to KASP or STARP markers for use in marker-assisted pre-breeding and breeding programs.

## Data Availability Statement

The datasets presented in this study can be found in online repositories. The names of the repository/repositories and accession number(s) can be found in the article/Supplementary Material.

## Author Contributions

ZM and AA performed field experiments. JM and MF performed the seedling experiment. AR, ZH, FO, and LH designed the experiment. RT conducted the 90K SNP genotyping. JP performed the GBS experiment. MA, AR, and KM analyzed the data. ZM, KM, and AR wrote the manuscript. All authors contributed to the article and approved the submitted version.

## Conflict of Interest

The authors declare that the research was conducted in the absence of any commercial or financial relationships that could be construed as a potential conflict of interest.

## Publisher’s Note

All claims expressed in this article are solely those of the authors and do not necessarily represent those of their affiliated organizations, or those of the publisher, the editors and the reviewers. Any product that may be evaluated in this article, or claim that may be made by its manufacturer, is not guaranteed or endorsed by the publisher.

## Footnotes

1
https://cran.r-project.org/web/packages/mrMLM/index.html
